# Blockade of EIF5A hypusination limits colorectal cancer growth by inhibiting MYC elongation

**DOI:** 10.1038/s41419-020-03174-6

**Published:** 2020-12-10

**Authors:** Sonia Coni, Silvia Maria Serrao, Zuleyha Nihan Yurtsever, Laura Di Magno, Rosa Bordone, Camilla Bertani, Valerio Licursi, Zaira Ianniello, Paola Infante, Marta Moretti, Marialaura Petroni, Francesca Guerrieri, Alessandro Fatica, Alberto Macone, Enrico De Smaele, Lucia Di Marcotullio, Giuseppe Giannini, Marella Maroder, Enzo Agostinelli, Gianluca Canettieri

**Affiliations:** 1 Department of Molecular Medicine, Sapienza University of Rome, Viale Regina Elena 291, 00161 Rome, Italy; 2 Department of Biochemical Sciences “A. Rossi Fanelli”, Sapienza University of Rome, Piazzale Aldo Moro 5, 00185 Rome, Italy; 3 Department of Biology and Biotechnologies “Charles Darwin”, Sapienza University of Rome, Piazzale Aldo Moro 5, 00185 Rome, Italy; 4 Center for Life Nano Science@Sapienza, Istituto Italiano di Tecnologia, Viale Regina Elena 291, 00161 Rome, Italy; 5 Department of Experimental Medicine, Sapienza University of Rome, Viale Regina Elena 324, 00161 Rome, Italy; 6 Cancer Research Center of Lyon (CRCL), UMR Inserm U1052/CNRS 5286, Lyon, France; 7 Istituto Pasteur, Fondazione Cenci-Bolognetti, Sapienza University of Rome, Viale Regina Elena 291, 00161 Rome, Italy; 8 International Polyamines Foundation-ONLUS, Via del Forte Tiburtino 98, 00159 Rome, Italy; 9 Department of Sense Organs, Sapienza University of Rome, Policlinico Umberto I, Viale del Policlinico 155, 00161 Rome, Italy

**Keywords:** Cancer therapy, Translation

## Abstract

Eukaryotic Translation Initiation Factor 5A (EIF5A) is a translation factor regulated by hypusination, a unique posttranslational modification catalyzed by deoxyhypusine synthetase (DHPS) and deoxyhypusine hydroxylase (DOHH) starting from the polyamine spermidine. Emerging data are showing that hypusinated EIF5A regulates key cellular processes such as autophagy, senescence, polyamine homeostasis, energy metabolism, and plays a role in cancer. However, the effects of EIF5A inhibition in preclinical cancer models, the mechanism of action, and specific translational targets are still poorly understood. We show here that hypusinated EIF5A promotes growth of colorectal cancer (CRC) cells by directly regulating MYC biosynthesis at specific pausing motifs. Inhibition of EIF5A hypusination with the DHPS inhibitor GC7 or through lentiviral-mediated knockdown of DHPS or EIF5A reduces the growth of various CRC cells. Multiplex gene expression analysis reveals that inhibition of hypusination impairs the expression of transcripts regulated by MYC, suggesting the involvement of this oncogene in the observed effect. Indeed, we demonstrate that EIF5A regulates MYC elongation without affecting its mRNA content or protein stability, by alleviating ribosome stalling at five distinct pausing motifs in MYC CDS. Of note, we show that blockade of the hypusination axis elicits a remarkable growth inhibitory effect in preclinical models of CRC and significantly reduces the size of polyps in APC^Min/+^ mice, a model of human familial adenomatous polyposis (FAP). Together, these data illustrate an unprecedented mechanism, whereby the tumor-promoting properties of hypusinated EIF5A are linked to its ability to regulate MYC elongation and provide a rationale for the use of DHPS/EIF5A inhibitors in CRC therapy.

## Introduction

Colorectal cancer (CRC) is third most deadly and fourth most commonly diagnosed cancer in the world^1^.

The disease typically begins as a benign adenomatous polyp, which develops into advanced adenoma with high-grade dysplasia and then to invasive cancer. In the early stages, cancer cells are confined within the wall of the colon and the disease can be cured by surgical excision. At later stages, cells cross the wall of the colon and reach the regional lymph nodes and CRC is curable in about 70% of cases with a combination of surgery and adjuvant chemotherapy. At final stage, the cells metastasize to distant sites and the prognosis becomes poor, with an average survival rate of 25–30 months with the current therapeutic approaches^2,3^. Despite the extensive characterization of the molecular alterations underlying CRC, the current therapeutic protocols include only a few drugs of limited efficacy and the overall survival rate is still unsatisfactory, especially in the advanced stages. For this reason, substantial effort is devoted at the identification of novel druggable molecular determinants of this disease, with the aim to identify suitable therapeutic targets.

Comprehensive molecular characterization of human CRC have led to the identification of genes and pathways that contribute to the initiation and progression of the disease.

The first molecular alteration occurring during colorectal tumorigenesis is a loss-of-function mutation of the *APC* gene, a genetic lesion found in the majority of CRCs that causes aberrant activation of the WNT-β-catenin pathway^4^. A germline mutation of the *APC* gene causes familial adenomatous polyposis (FAP), a genetic disorder characterized by hundreds of polyps in the large intestine that, if left untreated, progress toward malignant carcinomas^5–7^. Mutations of additional genes and pathways, such as RAS-MAPK, PI3K, TGFβ, P53, SMAD4, and DNA mismatch repair pathways, contribute to the progression of CRC toward the different stages^8^.

Integrative analysis of the molecular alterations has revealed that nearly all CRCs have changes in MYC transcriptional targets^9^, and that the deregulated pathways all converge on the activation of this oncogene. Hence, these observations underscore the critical pathogenic role played by MYC in CRC and imply that its targeting could represent a valuable therapeutic option. However, although direct inhibition of MYC is difficult because of its flat structure, indirect targeting of its degradation or biosynthesis has been challenging due to the multiple compensatory mechanisms that restore its intracellular content. An alternative pursued strategy is the targeting of MYC-regulated pathways that are required for tumor growth^10^. In this regard, inhibition of Ornithine decarboxylase (ODC), the first and rate-limiting enzyme in the polyamine biosynthesis pathway, and a direct MYC transcriptional target^11^ has been proposed as a potential therapeutic option in malignancies driven by the MYC oncogenes, such as lymphoma and neuroblastoma^10,12^.

ODC catalyzes the conversion of ornithine into putrescine (PUT), which is then converted into spermidine (SPD) and spermine (SPM). The three polyamines (PUT, SPD, and SPM) are often elevated in cancer and inhibition of their biosynthesis, through the irreversible ODC inhibitor difluoromethylornithine (DFMO), significantly impairs tumorigenesis in preclinical and clinical settings^13^. Of importance, DFMO has been shown to be a promising chemopreventive tool in subjects with high risk of CRC development, such as FAP patients^14^.

The major limitation to the use of DFMO for long-term treatments is that cells eventually become resistant to this drug, because they restore the intracellular polyamine pool by upregulating polyamine transporters and uptake from the extracellular environment^15^. Thus, to overcome this intrinsic limitation, a better approach would be the inhibition of key polyamine-regulated processes required for the tumor-promoting properties of these molecules. In this regard, recent studies are pointing at the link between polyamines and translation, and in particular to the translation factor (EIF5A), whose activity is strictly dependent on the polyamine levels. Two isoforms of EIF5A have been described in mammals: EIF5A1 and EIF5A2, both activated by hypusination, a unique covalent modification that requires SPD as substrate^16^. Indeed, the *n*-butylamine group of SPD is transferred to lysine group of EIF5A by the enzyme deoxyhypusine synthase (DHPS) to form deoxypusine, which is then hydroxylated to hypusine by deoxyhypusine hydroxylase (DOHH)^17,18^. Hypusinated EIF5A functions mainly by facilitating translational elongation of specific transcripts, by alleviating ribosome pausing at specific stalling motifs, often containing proline residues^19,20^. Recent studies have shown that EIF5A is overexpressed in various cancers, including CRC, where it correlates with poor prognosis^21^. Also, previous reports have documented the therapeutic efficacy achieved by inhibition of hypusination with the specific DHPS inhibitor GC7 in some cancers^22^. However, the actual mechanism of action and the direct translational targets of DHPS-EIF5A axis, responsible for the tumor-promoting effects, still remain largely unknown.

Using pharmacological and genetic inhibition approaches, in the present work we have studied the effect of DHPS-EIF5A axis inhibition on CRC growth and investigated the molecular mechanism of action. Our work has led to the identification of MYC as a key translational target of hypusinated EIF5A and to the demonstration of the therapeutic efficacy of targeting this molecular axis in vitro and in vivo.

## Results

### Inhibition of hypusination limits CRC cell growth

**Fig. 1:**
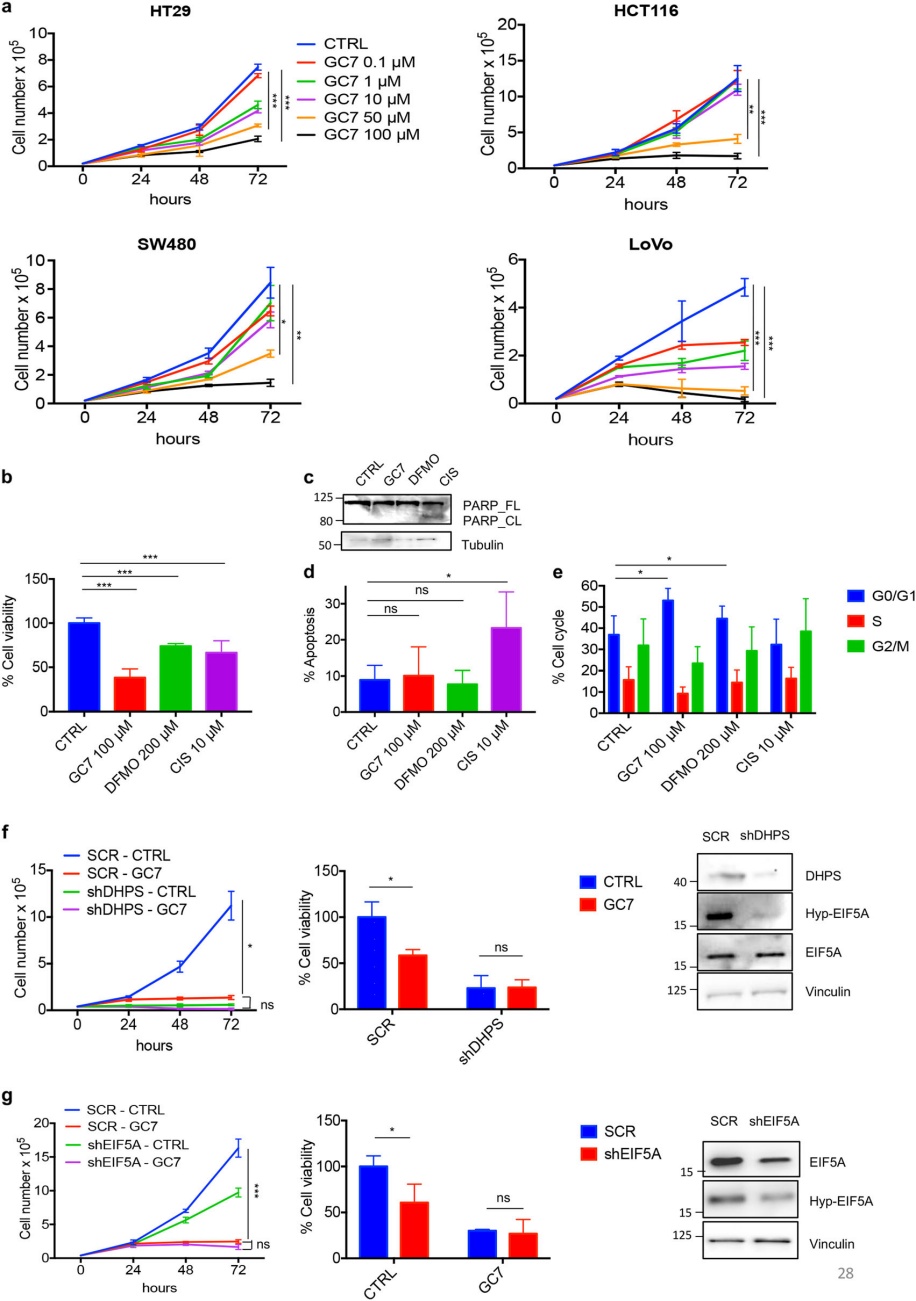
Inhibition of hypusination limits CRC cell growth in vitro. **a** Proliferation assay in HT29 (*n* = 3), HCT116 (*n* = 3), SW480 (*n* = 3), and LoVo (*n* = 3) cells treated with different concentrations of GC7 (0.1, 1, 10, 50, and 100 μM) for 24, 48, and 72 h (upper panels). **b** Cell viability assay (MTT) (*n* = 3) of HCT116 treated for 48 h with 100 μM GC7, 200 μM DFMO, 10 μM Cisplatin, or control vehicle. **c** Western blot analysis of full-length and cleaved PARP, tubulin (loading control) in HCT116 cells treated with 100 μM GC7, 200 μM DFMO, 10 μM Cisplatin, or control vehicle for 48 h. **d** Flow cytometry analysis (*n* = 3) of HCT116 cells stained with annexin V after 24 h from treatments with 100 μM GC7, 200 μM DFMO, 10 μM Cisplatin, or control vehicle. **e** Cell cycle profile (*n* = 3) of HCT116 cells treated with 100 μM GC7, 200 μM DFMO, 10 μM Cisplatin, or control vehicle for 24 h. **f** Left, HCT116 cells were infected with lentiviruses expressing DHPS (shDHPS) or control (SCR) shRNAs for 72 h. After infection, cells were incubated with 100 μM GC7 and counted at the indicated time points or treated with 100 μM GC7 for 48 h and MTT assay performed (middle). Right, western blotting showing DHPS, Hyp-EIF5A, EIF5A, and Vinculin (loading control) levels before treatment. **g** Left, HCT116 cells were infected with lentiviruses expressing EIF5A (shEIF5A) or control (SCR) shRNAs for 72 h. After infection, cells were incubated with 100 μM GC7 and counted at the indicated time points or treated with 100 μM GC7 for 48 h and MTT assay performed (middle). Right, western blotting showing EIF5A, Hyp-EIF5A, and Vinculin (loading control) levels before treatment. For statistical analysis: ns not significant, *p < 0.05, **p < 0.01, ***p < 0.001, by one-way ANOVA. Data represent the mean ± SD of experiments performed in triplicates and repeated at least three times.

We first studied the effect of the DHPS inhibitor GC7 on the growth of various human CRC cell lines (HT29, HCT116, SW480, and LoVo), characterized by distinct molecular alterations, typically found in the human disease^23^. As shown in Fig. 1a and Supplementary Fig. 1a–b, the drug inhibited proliferation and EIF5A hypusination at micromolar doses, being the standard 100 μM concentration very effective in all cells tested. At this concentration, the drug did not significantly change the intracellular content of the three polyamines (PUT, SPD, and SPM) after 72 h of treatment. Similar results were obtained with the irreversible ODC inhibitor DFMO (Supplementary Fig. 1c–d), which significantly inhibited EIF5A hypusination and tumor cell growth, demonstrating the dependence on polyamine metabolism for CRC growth.

MTT (3-(4,5-dimethylthiazol-2-yl)-2,5-diphenyltetrazolium bromide) assay showed the ability of GC7 and DFMO to decrease CRC cell viability (Fig. 1b), an effect that could not be attributed to apoptosis, as shown by the lack of poly (ADP-ribose) polymerase cleavage (Fig. 1c), as well as by the unmodified percentage of apoptotic cells observed by flow cytometry analysis of annexin V and propidium iodide double staining (Fig. 1d).

Instead, the two drugs caused inhibition of cell cycle, as documented by an increase of the percentage of cells in G0/G1 phase at the expense S and G2/M phases (Fig. 1e).

Therefore, these data indicate that pharmacological blockade of the hypusination axis significantly prevents proliferation of CRC cells, in agreement with previous reports^22,24–26^.

To ascertain that the antiproliferative effect of GC7 was not due to off target effects, we performed lentiviral-mediated knockdown of DHPS in HCT116 cells. Both DHPS and hypusinated EIF5A were disrupted in shDHPS-expressing cells (Fig. 1f, right panel) confirming the efficacy of the knockdown. Proliferation rate and viability of DHPS-deficient cells were strongly reduced compared to control cells (Fig. 1f) and GC7 failed to further inhibit cell viability in cells lacking DHPS, indicating the specificity of the drug (Fig. 1f). Similarly, knocking down EIF5A caused a significant decrease of CRC cell growth, and cell viability was no longer reduced by GC7 treatment in EIF5A-deficient cells (Fig. 1g).

Collectively, these results demonstrate that inhibition of polyamine-DHPS-EIF5A axis elicits significant antitumor effects in CRC cells.

### DHPS inhibition downregulates MYC

**Fig. 2:**
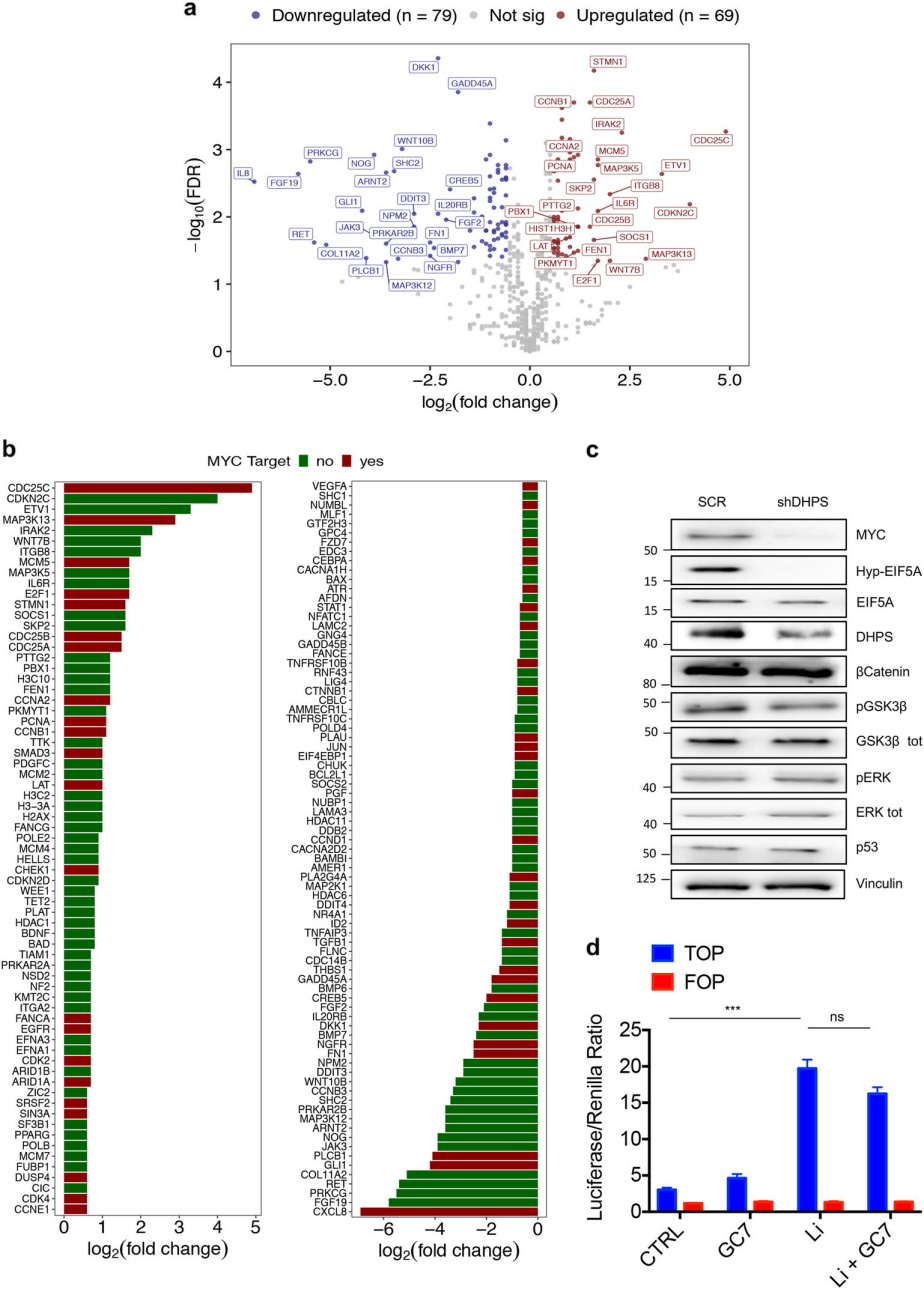
Gene expression profiling reveals that DHPS inhibition downregulates MYC. **a** Volcano plot displaying the 148 differentially expressed genes in shDHPS vs. SCR. Axes show logarithmic transformation of fold change (x-axis) and *p*-values (FDR). Genes significantly upregulated (*n* = 79) and downregulated (*n* = 69) with an FDR < 0.05 and a log2(fold change) < −0.58 or >0.58 are represented in red and in dark blue, respectively. Labeled points indicate the first 25 upregulated and first 25 downregulated genes. **b** Barplot showing the log_2_(fold change, shDHPS vs. SCR) of the significantly upregulated (left panel) or downregulated (right panel) genes. Red bars indicate MYC target genes. **c** Western blotting of HCT116 infected with lentiviruses expressing shDHPS or SCR for 72 h, then selected with puromycin for 72 h. Staining for MYC, Hyp-EIF5A, EIF5A, DHPS, β-Catenin, pGSK3β, GSK3β, pERK, ERK, and p53. Vinculin, loading control. **d** Luciferase assay (*n* = 3) in HCT116 cells transfected for 24 h with TCF/LEF responsive reporter TOP or its negative control FOP. The day after transfection, cells were serum starved for 8 h and treated with 50 mM lithium chloride and 100 µM GC7 for other 24 h. For Statistical analysis: ns not significant, *p < 0.05, **p < 0.01, ***p < 0.001, by Student’s *t*-test. Data represent the mean ± SD of experiments performed in triplicates and repeated at least three times.

To understand the mechanisms underlying the antitumor properties of DHPS inhibition, we performed NanoString-based gene expression analysis. We used the nCounter PanCancer pathway panel for gene expression, which monitors 730 genes from 13 cancer-associated hallmarks pathways, including those that are typically altered in CRC^27^. We analyzed mRNAs from DHPS-deficient or control CRC cells: 570 genes were detected, while the remaining 160 genes were not expressed. Within the expressed genes, 69 transcripts (12.2%) were upregulated, 79 (13.8%) were downregulated, whereas 422 (74%) were not modulated by the lack of DHPS (Fig. 2a and Supplementary Fig. 2). Notably, a relevant common regulator of the DHPS-modulated transcripts was the oncogene MYC, being 33% of the modulated mRNAs previously identified as transcriptional targets of this oncogene^28–32^ (Fig. 2b). Supporting these findings, immunoblotting analysis showed reduced levels of MYC (Fig. 2c) in cells lacking DHPS, compared to their control.

To determine whether the antitumor properties and reduced levels of MYC following DHPS inhibition could be related to alterations of one or more pathways commonly deregulated in CRC^32^ and able to impinge upon MYC function^9^, we analyzed the activation status of Wnt/β catenin, EGFR/MAPK, p53, and PI3K/AKT pathways in DHPS-depleted cells (Fig. 2c). Wnt/β catenin signaling, a pathway aberrantly activated in the majority of CRCs and a well-known regulator of MYC transcription^33^, was not affected by impaired hypusination, as shown by the unmodified β catenin protein levels and TCF/LEF-Luc reporter activity after DHPS inhibition (Fig. 2c–d). The levels of p53 were also not altered by DHPS inhibition as well as phosphorylated ERK and GSK3β, two markers of activation of the RAS-BRAF-MAPK and PI3K/AKT pathways, respectively (Fig. 2c).

Therefore, these data demonstrated that DHPS inhibition downregulates MYC levels independently of other CRC oncogenic drivers.

### Hypusinated EIF5A promotes MYC translation

To determine whether the observed MYC downregulation could be attributed to changes in mRNA levels, we performed quantitative PCR on CRC cells after DHPS inhibition.

**Fig. 3:**
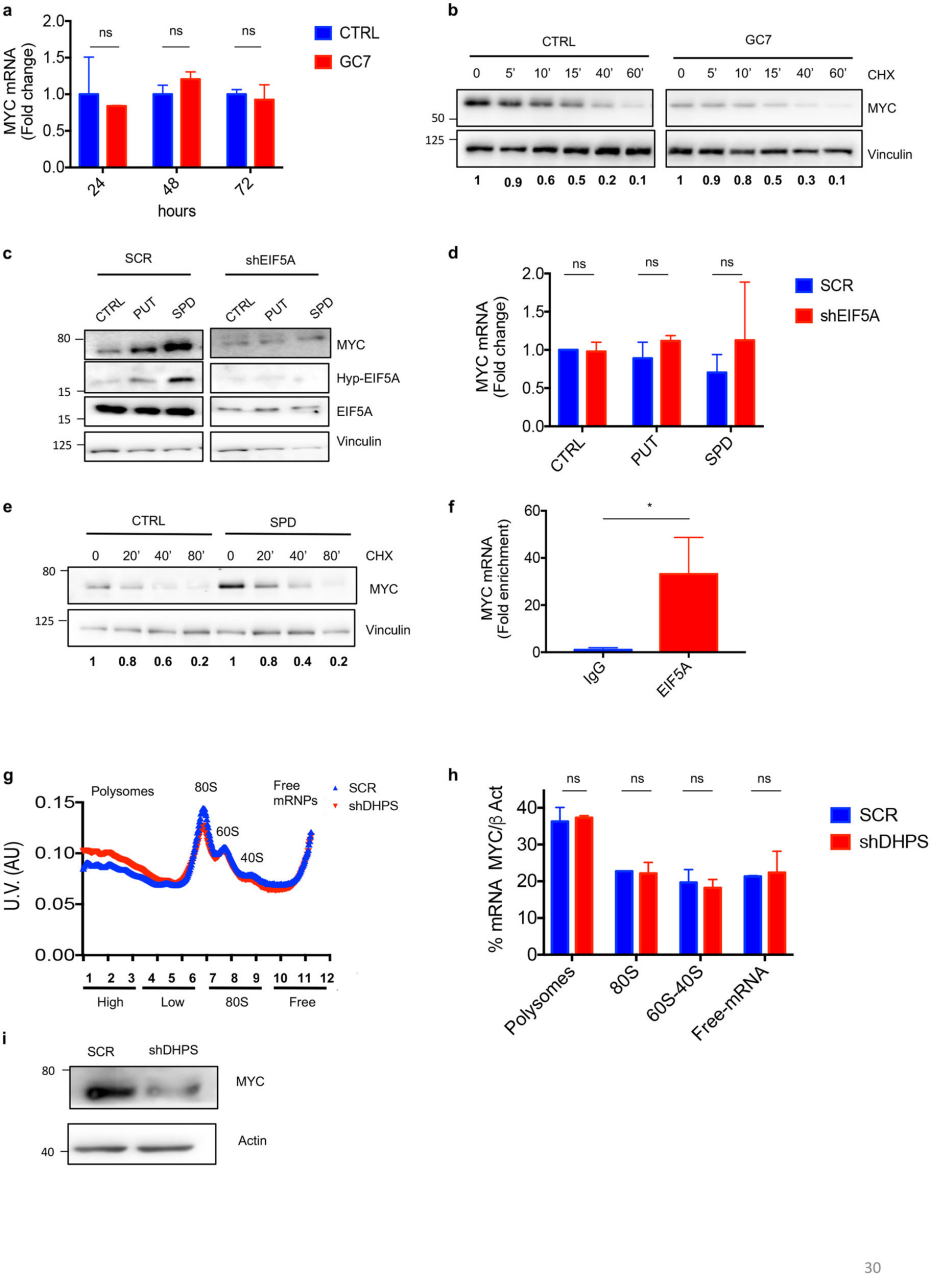
Polyamine-Hypusine axis regulates MYC translation in CRC. **a** Quantitative real-time PCR (*n* = 3) of MYC mRNA levels in HCT116 cells treated with 100 μM GC7 for the indicated times. Data were normalized using L32 mRNA. **b** HCT116 cells were treated with 100 µM GC7 or vehicle for 24 h and incubated with 100 μg/mL CHX for the indicated times. Immunoblottings were performed with the indicated antibodies. Vinculin, loading control. Densitometric analysis of MYC/Vinculin protein levels is shown at the bottom of each lane. **c** HCT116 cells were transduced with lentiviruses expressing shEIF5A or SCR for 72 h and selected with puromycin. Endogenous polyamines were depleted by incubating the cells with 1 mM DFMO for 3 days. Polyamine-depleted cells were treated with 10 μM Putrescine (PUT) or 10 μM Spermidine for 4 h. Immunoblottings were performed with the indicated antibodies. Vinculin, loading control. **d** MYC mRNA levels from **c**. Data were normalized by L32 mRNA; results are represented as fold change (*n* = 3). **e** HCT116 cells were depleted of polyamines as above and treated with 10 μM Spermidine (SPD) for 16 h. CHX (100 μg/ml) was added for the indicated time points. Staining for MYC protein levels is shown. Vinculin, loading control. Densitometric analysis of MYC/Vinculin protein levels is shown at the bottom of each lane. **f** RNA IP. HCT116 cells were crosslinked, lysed, and immunoprecipitated with antibody anti EIF5A or control IgG. After elution, MYC mRNA levels were analyzed by RT-QPCR. Data were normalized by L32 mRNA levels (*n* = 3). **g** Polysome profiles of DHPS-deficient and control (SCR) HCT116 cells. Cytoplasmic lysates were fractionated on 15–50% sucrose gradients. The graph shows ultraviolet (UV) absorbance at 260 nm of the different polysomal fractions (*n* = 3). **h** QPCR analysis of MYC mRNA loaded in the different polysome fractions, β-actin was used to normalize the values (*n* = 3). **i** Western blotting showing total MYC protein levels in the lysate used for polysomal fractionation. β-Actin, loading control. For Statistical analysis: ns not significant, *p < 0.05, **p < 0.01, ***p < 0.001, by Student’s *t*-test. Data represent the mean ± SD of experiments performed in triplicates and repeated at least three times.

As shown in Fig. 3a, GC7 treatment did not significantly change the levels of MYC mRNA compared to control cells, thus ruling out alterations of mRNA synthesis or turnover.

Moreover, DHPS inhibition did not modify MYC protein stability, as indicated by similar degradation rates in cells treated with the protein synthesis inhibitor cycloheximide (CHX) in the absence or presence of GC7 (Fig. 3b). Similar results were observed after short hairpin RNA (shRNA)-mediated knockdown of DHPS, being MYC mRNA levels and protein stability not modified by DHPS depletion (Supplementary Fig. 3a–b).

As these data suggested that DHPS-mediated hypusination is coupled to MYC translation, we next investigated whether the translational regulator EIF5A plays a direct role in this process. To this end, we first studied the effect of polyamine treatment on MYC protein levels, in the absence or presence of EIF5A. In analogy with other EIF5A-regulated translational targets (e.g., ODC or AZ1 and AZIN1)^34^, addition of PUT or SPD to polyamine-depleted media caused a marked increase of EIF5A hypusination and MYC protein, but not of MYC mRNA levels (Fig. 3c–d), and this upregulation was abrogated by the ablation of EIF5A, thus indicating the requirement of this translation factor. The effect of polyamines was not related to stabilization mechanisms, as witnessed by the unmodified turnover of MYC protein (Fig. 3e) after SPD administration.

Consistent with a role as a direct translational regulator, EIF5A was associated with MYC mRNA in CRC cells, as demonstrated by RNA immunoprecipitation (RIP) carried out with EIF5A antibody (Fig. 3f).

To specifically address if DHPS inhibition affects MYC translation, we analyzed ribosome loading of MYC mRNA in polyribosome-fractionated DHPS-deficient vs. control (SCR) CRC cells.

As shown in Fig. 3g, cells lacking DHPS showed polysome accumulation compared to control cells, an effect typically observed upon ribosome stalling^35^. By contrast, the content of MYC mRNA in the corresponding fractions was not significantly different (Fig. 3h), whereas MYC protein levels were reduced (Fig. 3i).

Collectively, these results support the hypothesis that inhibition of hypusination causes an increased ribosome stalling, thereby reducing MYC biosynthesis.

### EIF5A regulates MYC translation at tripeptide pausing motifs

**Fig. 4:**
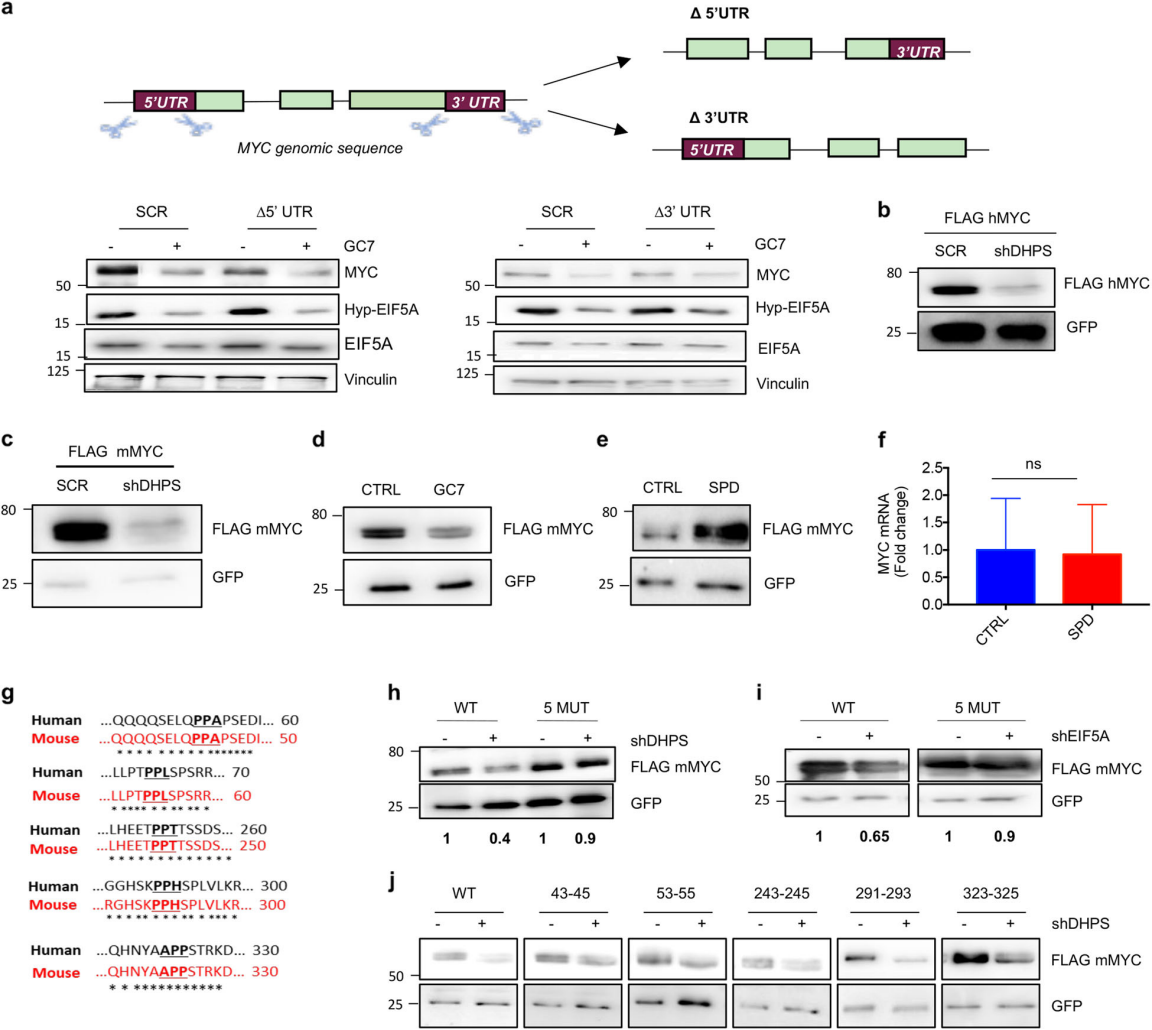
EIF5A regulates MYC translation at PPA sites in the coding region. **a** Schematic representation of CRISPR/Cas9 strategy used to delete 5′- or 3′-UTRs from MYC gene in HCT116 cells (Δ5′UTR and Δ3′UTR clones, respectively). Western blotting of MYC, Hyp-EIF5A, EIF5A, and Vinculin (loading control) in scrambled (SCR) and Δ5′UTR clones (left) or in SCR and Δ3′UTR (right) clones treated with 100 μM GC7 or vehicle for 24 h. **b** Western blotting using FLAG and GFP antibody in HCT116 cells infected with SCR or shDHPS lentiviruses and co-transfected with vectors expressing FLAG hMYC and GFP. **c** Western blotting using FLAG and GFP antisera in HCT116 cells infected with SCR or shDHPS lentiviruses and co-transfected with vectors expressing FLAG mMYC and GFP. **d** Western blot analysis of FLAG and GFP in HCT116 cells transfected with FLAG mMYC and GFP vectors and treated with 100 µM GC7 for 48 h. **e** Immunoblot analysis of FLAG and GFP co-transfected with FLAG mMYC and GFP vectors, in polyamines-depleted HCT116 cells for 3 days by using 1 mM DFMO incubation and then treated with 10 μM Spermidine for the last 16 h. **f** MYC mRNA levels from **e**. Data were normalized by β-actin mRNA (*n* = 3). **g** Alignment of human (black) and mouse (red) MYC aminoacidic sequences at the identified pausing motifs. **h** Western blot analysis of FLAG and GFP in HCT116 cells infected with shDHPS or SCR and transfected with FLAG mMYC WT or mMYC 5 MUT and GFP as control for 24 h. Densitometric analysis of MYC/GFP protein levels is shown at the bottom of each lane. **i** Immunoblot with FLAG and GFP antisera in EIF5A-deficient (shEIF5A) or control (SCR) HCT116 cells co-transfected for 24 h with plasmids expressing GFP and FLAG mMYC, WT or mutated in the putative five pausing motifs (mMYC 5 MUT). Densitometric analysis of MYC/GFP protein levels is shown at the bottom of each lane. **j** Immunoblot with FLAG and GFP antisera in DHPS-deficient (shDHPS) or control (SCR) HCT116 cells co-transfected for 24 h with plasmids expressing GFP and FLAG mMYC, WT or mutated in the indicated individual pausing motifs.

As EIF5A has been described to regulate translation through multiple mechanisms^34^ potentially acting at different mRNA levels, we next sought to identify the mRNA region required for MYC translational regulation. To this end, using the CRISPR/Cas9 approach we generated ∆5′UTR-MYC and ∆3′UTR-MYC CRC cells carrying deletions of MYC 5′- or 3′-untranslated regions (UTRs), respectively (Fig. 4a upper panel). Inhibition of hypusination with GC7 continued to cause a decrease of MYC protein levels in both ∆5′UTR-MYC and ∆3′UTR-MYC CRC cells (Fig. 4a bottom left and right, respectively), indicating that the regulation does not require MYC UTRs but rather its coding region. In agreement with this hypothesis, DHPS inhibition caused a significant decrease of exogenous MYC protein, encoded by plasmids expressing human or mouse MYC coding region, but lacking UTRs (Fig. 4b–d). Similarly, SPD supplementation induced exogenous MYC protein, but not its mRNA levels (Fig. 4e–f), indicating that EIF5A promotes MYC translation by acting at the coding region.

Previous work demonstrated that EIF5A alleviates translational pausing of ribosomes at specific stalling motifs in yeast^19^. In particular, through ribosome-profiling screening, it was demonstrated that the absence of EIF5A causes stalling of ribosomes at the level of 29 different tripeptide motifs, which may or may not contain proline residues^20,36^. We analyzed the amino acid sequence of both human and mouse MYC CDS and found the presence of 5 potential stalling sites, located in mouse at aa positions: 43–45, 53–55, 243–245, 291–293, and 323–325, and conserved in human (Fig. 4g).

Mutation of all these five pausing sites (MYC 5 MUT) prevented the decrease of MYC protein levels caused by DHPS inhibition (Fig. 4h) or EIF5A ablation (Fig. 4i), confirming their involvement in the observed effect. By contrast, individual mutations of each of these motifs did not modify the inhibition of MYC protein levels after DHPS ablation (Fig. 4j and Supplementary Fig. 4a), indicating that multiple sites are required for the inhibitory effect.

### Effect of DHPS inhibition in preclinical models of CRC and FAP

**Fig. 5:**
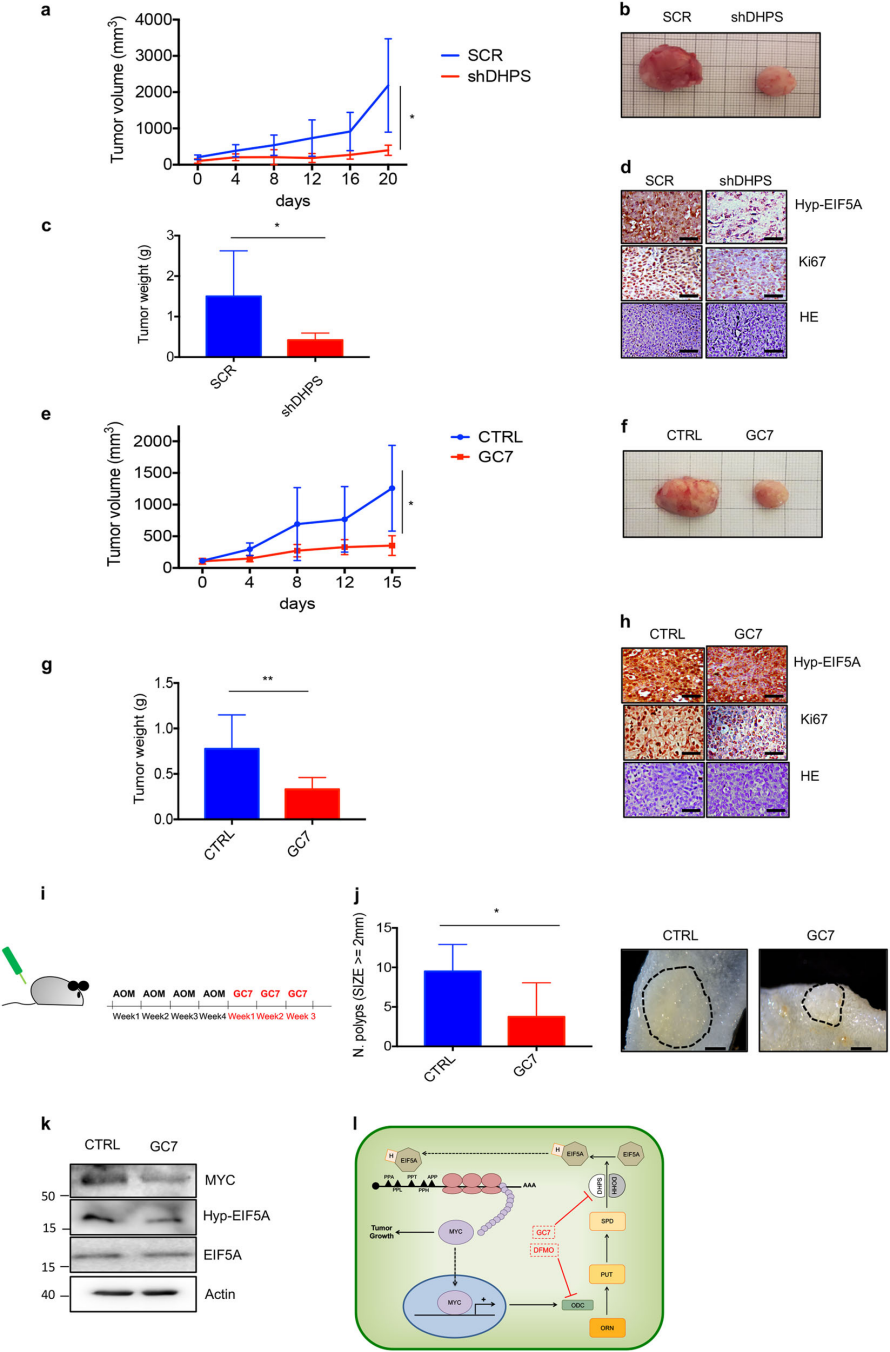
Pathophysiological relevance of DHPS inhibition in preclinical models of CRC and FAP. **a** HCT116 cells were stably transduced with lentiviruses expressing DHPS (shRNA) or nonspecific shRNAs (SCR) and then implanted subcutaneously into CD1 nude mice (2 × 10^6^ cells each flank). Tumors were measured starting when they reached an average volume of 100 mm^3^. (SCR *n* = 6; shDHPS *n* = 6). **b** Representative images of explanted masses from **a** at the end of the procedure. **c** Average tumor weight of the explanted masses from **a**. **d** Hyp-EIF5A, Ki67, or hematoxylin staining of tumor sections from explanted masses shown in **b**. Scale bar 100 μM. **e** HCT116 cells were implanted subcutaneously into CD1 nude mice (2 × 10^6^ cells each flank). When tumors reached an average volume of 100 mm^3^, mice were treated i.p. with GC7 4 mg/kg or control vehicle. Tumor volumes were measured at the indicated days. (CTRL *n* = 8; GC7 *n* = 8). **f** Representative images of explanted masses from **e** at the end of the experiment (CTRL vs. GC7). **g** Average tumor weight of the explanted masses from **e** at the end of the procedure. **h** Hyp-EIF5A, Ki67, or hematoxylin staining of tumors from **f**. Scale bar 100 μM. **i** Protocol of AOM (12 mg/kg) and GC7 (25 mg/kg) treatments in APC^Min/+^ mice. **j** Left, number of polyps ≥ 2 mm in size in the small intestine of female APC^Min/+^ mice treated with GC7 (*n* = 3) or CTRL (*n* = 3) as shown in **i**. Right, representative image of the polyps. Scale bar 1 mm. **k** Western blot analysis of MYC, Hyp-EIF5A, EIF5A, and β-actin (loading control) levels in pooled extracts from polyps in the small intestine of APC^Min/+^ mice treated as in **i**. Control, *n* = 3; GC7, *n* = 3. Schematic Model. Hypusinated EIF5A (Hyp-EIF5A) promotes MYC elongation by alleviating ribosome stalling at five pausing motifs. By regulating transcription of the enzyme Ornithine decarboxylase (ODC), MYC promotes an increase of the three polyamines (PUT, SPD, SPM). Elevations of SPD content lead to increased Hyp-EIF5A, thereby sustaining an amplification feedback loop. Inhibition of this mechanism limits intestinal tumor growth.

To determine the pathophysiological relevance of the DHPS-EIF5A axis in CRC, we first evaluated the effect of DHPS ablation on the growth of human CRC cells grafted into the flanks of athymic nude mice. DHPS-deficient CRC cells grew significantly slower then controls and at the end of the experiment tumor sizes, volumes, and weight of the explanted masses were greatly reduced compared to controls (Fig. 5a–c). Ki67 staining and EIF5A hypusination were markedly reduced in cells depleted for DHPS compared to the controls (Fig. 5d).

To evaluate the therapeutic benefit of pharmacological DHPS blockade in mouse tumor models, we studied the effects of GC7 treatment on the growth of grafted human CRC cells. We implanted HCT116 cells subcutaneously in nude mice and when the tumor volume reached 100 mm^3^, mice were intraperitoneally (i.p.)-injected daily with GC7 or phosphate-buffered saline (PBS) for 15 days and the growth of tumor volumes monitored every 4 days. Treatment with GC7 significantly decreased tumor growth and caused a significant reduction of average tumor volume and weight compared to control (Fig. 5e–g), accompanied to a strong reduction of Ki67 staining and EIF5A hypusination (Fig. 5h).

To address the efficacy of DHPS inhibition on spontaneous intestinal tumorigenesis, we investigated the effect of GC7 treatment in APC^Min/+^ mice. This mouse model carries heterozygous inactivating mutation of *APC* allele, which is associated to multiple intestinal neoplasms, a phenotype reminiscent of human FAP^37^.

Loss-of-function mutation of *APC* gene in this mouse model causes aberrant activation of the Wnt/β catenin pathway with consequent upregulation of MYC, which plays a key role in the development of this disease^4,33^.

APC^Min/+^ mice were weekly injected with AOM (Azoxymethane) for 1 month to induce neoplasms and then treated with daily i.p. injections of GC7, for a total of 3 weeks (Fig. 5i). At the end of the treatment, mice were killed and the intestines explanted and analyzed. As shown in Fig. 5j (left panel), GC7 treatment significantly impaired the growth of intestinal polyps, resulting in a marked decrease of the size of the lesions (Fig. 5j right panel). The effect of the drug was associated to a decrease of MYC protein levels and hypusinated EIF5A in the analyzed polyps (Fig. 5k), confirming the efficacy of the treatment.

Taken together, these data demonstrate that inhibition of EIF5A hypusination provides therapeutic benefit in preclinical models of CRC and FAP.

## Discussion

The involvement of elevated intracellular polyamines in colorectal tumorigenesis has been known for many years and many reports have documented the ability of the ODC inhibitor DFMO to limit tumor formation and progression in preclinical models, as well as its chemopreventive efficacy in clinical trials^13^. However, the effect of DFMO is transient, mainly because of the very short half-life of ODC protein and tight regulation of its biosynthesis and because cells reconstitute polyamine content by increasing the uptake from the extracellular environment, through upregulation of polyamine transporters^13^. Hence, there is an increasing interest for the identification of drugs that have the ability to stably reduce intracellular polyamine content or inhibit polyamine target/s involved in pathological processes regulated by these molecules.

Emerging evidence are showing that the translational regulator EIF5A mediates many of the known effects of polyamines, such as the regulation of cell proliferation, viability, migration, and autophagy^16,38,39^. Also, many studies show increased levels of EIF5A in tumor cells^22^. In particular, a previous report showed that EIF5A is overexpressed in CRC tissues, and that its elevated expression is associated to poor prognosis^21^. However, to date, only few reports have documented the effect of EIF5A inhibition on in vivo tumor growth and it is not clear which translational targets mediate the EIF5A effect.

In the present work, we have shown for the first time that pharmacological or genetic inhibition of EIF5A hypusination efficiently inhibits the growth of CRC cells in vitro and in vivo. Of note, the DHPS inhibitor GC7 showed a remarkable efficacy in limiting the growth of CRC cells and polyps in mice.

The use of GC7 as anticancer agent in animal models has been studied in a limited number of tumors. In particular, previous work showed that in KRas-mutated pancreatic ductal adenocarcinoma cells, EIF5A1-PEAK axis positively regulates KRas neosynthesis, thus enhancing ERK-MAPK signaling^40^ and promoting cancer growth and invasion. KRas in turn promotes EIF5A protein expression, thus generating a positive feed forward loop that promotes glutamine-dependent cancer cell growth. Notably, disruption of this mechanism with the DHPS inhibitor GC7 limits pancreatic tumor growth in vivo and sensitizes tumor cells to the treatment with MEK inhibitors^40^. Although the authors did not formally demonstrate that KRas is a direct translational target of EIF5A, this report provided a mechanistic explanation for the tumor-promoting effect of EIF5A in this type of malignancy.

In contrast with these studies, our data show that inhibition of CRC cells does not modify ERK content and phosphorylation, thus supporting the conclusion that EIF5A function is not coupled to KRas expression and activity in intestinal tumors.

Conversely, our data indicate that inhibition of polyamine metabolism and EIF5A hypusination suppresses MYC protein levels, regardless to KRas mutational status, being the effect observed also in HT29 CRC cells, where *KRas* allele is not mutated^23^ (Supplementary Fig. 4b).

The finding that MYC is downregulated by GC7 is of particular relevance, as targeting of this oncogene represents a challenging option in cancer therapy and as previous attempts to inhibit this oncogene in CRC with clinically approved translational inhibitors, such as mammalian target of rapamycin inhibitors, have failed^41^.

A link between polyamines and MYC biosynthesis was previously described in rat intestinal epithelial cells by Liu and collaborators. In particular, it was observed that polyamines regulate MYC biosynthesis by increasing Chk2-mediated phosphorylation of the mRNA binding protein HuR and its association to the 3′-UTR of MYC, thereby promoting its translation^42^. By contrast, our data seem to rule out the possibility that such a mechanism could operate also in our model since inhibition of DHPS still suppresses MYC translation in CRC cells deleted of MYC 3′-UTR. Instead, our data show the involvement of MYC coding region in this process, thus supporting the interpretation that EIF5A mediates translational elongation. In this context, a major function of EIF5A seems to be the suppression of ribosome pausing within the coding region occurring at specific pausing motifs, thereby facilitating elongation^19,20^.

Interestingly, a recent work by Manjunath et al.^43^ showed that EIF5A controls the start codon selection of mRNAs containing initiation codons upstream of the canonical ATG sequence, including MYC. Indeed, MYC translation can be initiated at either canonical ATG or upstream CTG codons, giving rise to two different isoforms: MYC2 (short, 64 kDa) and MYC1 (long, 67 kDa), respectively. The authors showed that, by alleviating ribosomal pausing at a single upstream tripeptide proline-proline-alanine (PPA) pausing motif, located between the two start codons, EIF5A regulates the start codon selection of MYC and favors the initiation at canonical ATG codon. However, the impact of this mechanism in tumorigenesis and its conservation throughout the species was not addressed in that report.

Our data indicate that inhibition of EIF5A hypusination reduces global levels of MYC in human and mouse cells, and causes inhibition of tumor growth.

Curiously, protein alignment of the N-terminal regions of MYC shows that the PPA motif and the surrounding region found in human^43^ are not conserved in mouse, suggesting a species specificity for the previously described mechanism. Of importance, our data indicate that both isoforms of MYC protein are downregulated by inhibition of EIF5A hypusination (Supplementary Fig. 4c–d), and that multiple pausing sites conserved between human and mouse CDS (Fig. 4g) are involved in the regulation. Therefore, our data support the conclusion that EIF5A plays a key role in controlling elongation of both MYC isoforms, by alleviating ribosome stalling at multiple common pausing sites.

Interestingly, a very recent work showed that GC7 treatment also inhibits p53 synthesis^44^. By contrast we did not see changes of p53 levels in CRC cells after shRNA-mediated DHPS ablation, suggesting that the observed effect could be related to the drug or the conditions used rather then to exclusive DHPS inhibition.

In conclusion, we have identified a novel mechanism whereby polyamines regulate MYC elongation through hypusinated EIF5A, by acting at specific pausing motifs. As MYC is also a key regulator of polyamine biosynthesis through *ODC* transcriptional activation, these findings imply that polyamines and MYC content are part of an integrated amplification loop that is aberrantly activated in tumors. Importantly, we have demonstrated that inhibition of this hypusination-dependent mechanism elicits significant therapeutic effects in preclinical models of CRC and FAP, thus representing a novel potential actionable target in these disorders (Fig. 5i). Further studies are required to establish the pharmacological properties of GC7 and its possible use in patients, alone or in combination with other drugs.

## Materials and methods

### Polysomal fractionation

For polysome fractionation, cells were treated with 100 μg/ml CHX for 5 min, washed twice with PBS solution, and lysed with 450 µl of TNM lysis buffer (10 mM Tris-HCl pH 7.4 or 7.5, 10 mM NaCl, 10 mM MgCl_2_, 1% Triton X-100) supplemented with 10 mM dithiothreitol, 100 μg/ml CHX, 1× PIC (#1187358001 complete, EDTA free, Roche, Mannheim Germany), and RiboLock RNase inhibitor (#EO0382 Thermo Fisher Scientific, Burlington, ON, Canada).

Lysates were incubated on ice for 10 min and then centrifuged. Supernatants were collected, loaded onto a 15–50% sucrose gradient, and ultracentrifugation was performed at 37,000 r.p.m. for 120 min with a SW41 rotor (Beckman Coulter, Brea, CA).Fractions collection was performed using Biorad-BioLogic LP/2110 (Hercules, California,USA), according to the manufacturer’s protocol. RNA extraction and real-time quantitative PCR were performed as previously described^45–47^.

### RNA immunoprecipitation

RIP was performed as described previously^48^. HCT116 cells were plated in 56 cm^2^ tissue culture dishes and 24 h later cells were crosslinked with 1% formaldehyde solution for 10 min at room temperature, and then incubated with glycine (125 mM) for 5 min at room temperature. Pellets were lysed with FA Buffer (50 mM HEPES pH 7.5, 140 mM NaCl, 1 mM EDTA, 1% Triton X-100, 0.1% sodium deoxycholate, protease inhibitors (1 mM phenylmethylsulfonyl fluoride,10 µg/ml leupeptin, 1 µg/ml pepstatin, 1 µg/ml aprotinin), 50 U/ml RNase inhibitor SupeRNase, #AM 2694 Thermo Fisher Scientific) and sonicated. Samples were treated with DNase (Invitrogen, Thermo Fisher Scientific) and 25 mM MgCl_2_, 5 mM CaCl_2_, and SupeRNAse (AM 2694, Thermo Fisher Scientific) for 10 min at 37 °C. The reaction was interrupted with the addition of 20 mM EDTA. Immunoprecipitation was performed incubating the samples with anti EIF5A antibody overnight as indicated in the Supplementary Table 2. The following day, protein A-agarose beads, previously incubated overnight with 200 µl FA buffer, 1 mg/ml salmon sperm DNA (#AM9680, Thermo Fisher Scientific), and RNAse Inhibitor were added to the lysate for 1 h at 4 °C in agitation; samples were washed extensively with four different solutions: low salt: 0.1% SDS, 1% Triton X-100 2 mM, EDTA 20 mM Tris-HCl pH 8, 150 mM NaCl, and 0.005 U/ml SuperRNAse (Thermo fisher Scientific); high salt: 0.1% SDS, 1% Triton X-100, 2 mM EDTA, 20 mM Tris-HCl pH 8, 500 mM NaCl, and 0.005 U/ml SuperRNAse; LiCl buffer: 0.25 M LiCl, 1% NP40, 1% sodium deoxycholate, 1 mM EDTA, 10 mM Tris-HCl pH 8, and 0.005 U/ml SuperRNAse; TE wash buffer: 10 mM Tris-HCl pH 8, 1 mM EDTA, and 0.005 U/ml SuperRNAse. Elution was performed with Elution buffer: 1% SDS, 0.1 M NaHCO_3_, SuperRNase 50 U/ml twice, and for 15 min at room temperature. RNA extraction was performed using Trizol reagent Thermo Fisher (#15596026). cDNA was obtained using SensiFAST™ cDNA Synthesis Kit (Bioline, London UK) and then analyzed by real-time PCR, with the indicated oligos.

### Animal studies

Xenograft studies were performed as previously described^49,50^. Briefly, 2 × 10^6^ HCT116 cells were resuspended in 50 µl PBS solution and mixed with equal volume of Matrigel (#354248 Corning, Corning NY, USA) and implanted subcutaneously in both flank of 8 weeks adult athymic nude mice (Charles River Laboratories Wilmington, MA, USA). Animals were divided in two groups (control: *n* = 8; GC7 treated: *n* = 8). When the average tumor volumes reached 100 mm^3^, mice were daily treated with 4 mg/kg GC7 i.p. and tumor sizes were measured every 4 days using a caliper.

For xenograft studies with DHPS-depleted cells, HCT116 cells were transduced with lentiviruses expressing shRNA targeting DHPS or nonspecific shRNA, as described above. Cells (2 × 10^6^) cells were implanted subcutaneously in both flanks of 8-week-old adult athymic nude mice. Animals were divided in two subgroups (SCR: *n* = 6; shDHPS: *n* = 6). Measurements began when tumor volume reached 100 mm^3^ and were measured every 4 days using a caliper.

Volume was calculated as *V* = (*L* × *W*^2^)/2. Growth patterns were summarized graphically by plotting the mean and SD for each treatment group over time.

For in vivo treatments of APC^Min/+^ mice, 12-week-old APC^Min/+^ female mice were treated once a week with AOM (A5486, Sigma Aldrich, St. Louis, MO USA) and 12 mg/kg for 4 weeks, through i.p. injections. Mice were then i.p.-injected with 25 mg/kg GC7 for a total of 3 weeks and then killed. Intestines were explanted, cleaned, and washed in PBS. Polyp sizes were measured manually by using a caliper. Animal studies were performed according to the European Community Council Directive 2010/63/EU and were approved by the local Ethical Committee for Animal Experiments of the Sapienza University of Rome (Aut. n.877/2016-PR).

### Statistical analysis

All experiments were performed multiple times and, where indicated, in triplicate to reach statistical significance, as specified in the figure legends.

Statistical analysis was performed using GraphPad Prism version 7.0 for Mac. Data were analyzed with a paired Student’s *t*-test, or analysis of variance and expressed as mean of three separate experiments ± SD. Data with *p < 0.05 were considered statistically significant.

## Supplementary information

Supplemental material

Supplementary Table 1

Supplementary table 2

Supplementary table 3

Supplementary table 4
