# Supplementary material for: Blockade of EIF5A hypusination limits colorectal cancer growth by inhibiting MYC elongation
Source: Cell Death Dis. 2020 Dec 10;11(12):1045. doi: 10.1038/s41419-020-03174-6 (PMC7729396; doi:10.1038/s41419-020-03174-6)

Blockade of EIF5A hypusination limits Colorectal Cancer growth by inhibiting MYC elongation

Sonia Coni, Silvia Maria Serrao, Zuleyha Nihan Yurtsever, Laura Di Magno, Rosa Bordone, Camilla Bertani, Valerio Licursi, Zaira Ianniello, Alessandro Fatica, Alberto Macone, Paola Infante, Marta Moretti, Marialaura Petroni, Francesca Guerrieri, Enrico De Smaele, Lucia Di Marcotullio, Giuseppe Giannini, Marella Maroder, Enzo Agostinelli and Gianluca Canettieri

Correspondence to: Gianluca Canettieri, MD, PhD
Sapienza University of Rome
Department of Molecular Medicine,
Viale Regina Elena 291 – 00161 Rome, Italy
Email: gianluca.canettieri@uniroma1.it
phone number: +39 06 4925 5130

This file contains

Supplementary Materials and Methods

Supplementary Figures 1-4

SUPPLEMENTARY MATERIALS AND METHODS

Cell cultures and drug treatments

HT29, SW480, LoVo and HCT116 colorectal cancer cells were cultured in Dulbecco's Modified Medium (DMEM, Sigma Aldrich) with 10% fetal bovine serum (FBS), 1% antibiotics (100 U/ml penicillin and 100 U/ml streptomycin) and 1% glutamine. All cell lines used were confirmed mycoplasma-free by PCR testing.

Drug treatments were performed using 100 μM, 200 μM, 500 μM, 1 mM and 5 mM DFMO (Sigma-Aldrich #D193) or 0.1 μM, 1 μM, 10 μM, 50 μM and 100 μM GC7 (#G-1000-10 LGC Biosearch technology London UK), for 24, 48 and 72 hours. 100 μg/ml Cycloheximide (CHX) (#C7698 Sigma-Aldrich) was added to HCT116 cells for the indicated times. 10 μM of both spermidine (SPD) or putrescin (PUT) (#S2626 and #51799 Sigma-Aldrich) were added to cells depleted of polyamines through a pre-treatment of 72h with 1 mM DFMO. 10 μM Cisplatin (#232120 Sigma-Aldrich) was used as a positive control for MTT assay. All drugs were added in DMEM medium.

Plasmids and antibodies

Lentiviral plasmids for RNA interference and CRISPR/Cas9-mediated deletion were generated in pLKO.1 vector (#8453 Addgene, Watertown, MA) and pLentiCRISPR-v2 (#52961 Addgene plasmid) respectively. The oligonucleotides and sgRNA sequences are described in Supplementary Table 1 and 3.

Human Flag-MYC was purchased from Addgene (#102625) and mouse Flag-MYC was kindly provided by Drs Sergio Nasi and Barbara Illi (CNR, Rome, Italy). Mouse Flag-MYC plasmid was used as a template to generate mutants for the polyproline sites (single or multiple mutant) described in the main text. Reporter vectors M50 Super 8x TOP-Flash (#12456 Addgene), M51 Super 8x FOP-Flash (#12457 Addgene) and TK renilla[1] were used for luciferase assay.

The antibodies used are listed in Supplementary Table 2.

Transfections and proliferation assays

HCT116 were transfected using 4 µl/µg DNA of DreamFect transfection reagent (#DF41000 OZ biosciences, Marseille, France) according to the manufacturer's protocol.

Proliferation assays were performed plating 10^4 or $2x10^4$ cells/cm^2 and counting their number with a Burker chamber at the indicated time points, after staining with TrypanBlue (#T6146 Sigma-Aldrich).

CRISPR/Cas9-mediated deletion of MYC 5' and 3' UTRs

MYC 5' and 3' untranslated regions were deleted using short guide RNA (sgRNA) targeting the regions of interest. sgRNA were designed using the Benchling online tool (https://www.benchling.com/). The sequences are described in Supplementary Table 3. Complementary oligonucleotides were annealed and inserted into pLentiCRISPR-v2 (Addgene plasmid #52961). For transient expression of Cas9 and sgRNAs, HCT116 cells were transfected with the Δ5'UTR-MYC or Δ3'UTR-MYC constructs and selected with puromycin (#ALX-380-028. EnzoLifeScience) for 72 hours, then diluted to allow clonal growth, collected and screened by PCR using the primers described in Supplementary Table 3.

Western blot

For western blotting, cells were lysed in denaturing buffer SDS-urea (50 mM TrisHCl pH 7.8, 2% SDS, 10% glycerol, 10 mM $Na_4P_2O_7$, 100 mM NaF, 6 M urea, 10 mM EDTA). Protein extracts were then sonicated, quantified, resolved by SDS–polyacrylamide gel electrophoresis and transferred to a nitrocellulose membrane (#NBA085C001EA, Perkin Elmer, Waltham, MA, USA).

Membranes were blocked with 5% milk in Tris buffered saline with 0.1% Tween 20 and incubated

in the same buffer with primary antibodies overnight (or with HPR-conjugated primary antibodies for 1 hour) and HPR-conjugated secondary antibodies. Detection of the horseradish peroxidase signal was performed using WesternBright ECL (#K-12045-D50, Advansta, San Jose, CA, USA). Densitometric analysis was performed using ImageJ program (version 1.50i)

Luciferase assay

HCT116 cells were seeded $2x10^4$ cells/cm^2 in 24 well plates in triplicate. The following day, cells were transfected using DreamFect transfection reagent, according to the manufacturer's protocol, with 50 ng reporter vector M50 Super 8x TOPFlash or 50 ng control reporter vector M51 Super 8x FOPFlash. All reports were co-transfected with 20 ng TK renilla vector. After 24 hours the cells were incubated with starvation medium (Optimem, FBS 0.5%, penicillin 1%, streptomycin 1%, sodium pyruvate 1%, nonessential amino acids 1%) for 8 hours. After starvation, cells were treated with Lithium chloride (50 mM) (#203637 Sigma-Aldrich) for 24 hours to activate the WNT/beta-chain pathway. The following day, cells were treated with 100 µM GC7 for 24h. Cell extracts were prepared with lysis Buffer (passive lysis buffer 5x, #99912 Biotium, Fremont, CA, USA) according to the manufacturer's protocol; D-Luciferine (#10101 Biotium) was diluted in the noncommercial stock luciferase assay buffer as described[2] with a final concentration of 40 µg/ml and coelenterazine (#S053 Synchem) was diluted in PBS. Luminescence was read with Glomax Explorer Multimode Microplate Reader (#GM3500, Promega, Medison, WI, USA). Relative luciferase activity is expressed as the ratio of luciferase and renilla activity in control and treated cells expressing TOP and FOP plasmids.

RNA extraction, retro-transcription and qPCR

Total mRNA was isolated from cells with the Trizol reagent (#15596026, Thermofisher) according to the manufacturer's instructions. 1 µg of total RNA was reverse-transcribed with SensiFAST™ cDNA Synthesis Kit (#BIO-65053 Bioline). Real-time PCR was performed using

SensiFast Sybr Lo-Rox Mix (#BIO-94020, Bioline) and transcript levels were quantified with the Applied Biosystems (Waltham, Massachusetts, USA) ViiA 7 Real-Time PCR System 36 instrument. The primers used are listed in Supplementary Table 4.

Lentivirus - mediated shRNA Knockdown

Lentivirus production was performed as described[3]. Virus titers were determined using quantitative real-time PCR as previously described[4]. To perform lentiviral transduction, HCT116 cells were seeded at a density of $2x10^4$cell/cm^2 and transduced with lentiviruses (MOI=5) and polybrene at final concentration of 5 µg/ml (#H9268, Sigma-Aldrich) for 72h. After 72h infected cells were selected with 5 µg/ml of puromycin (#ALX-380-028. EnzoLifeScience, Farmingdale, New York, USA) for 72h. Knockdown efficiency was monitored by western blotting.

Nanostring-based gene expression analysis

HCT116 cells were infected with PLKO.1 lentiviral vector expressing shRNA targeting DHPS enzyme or non-specific shRNA (PLKO.1 control vector), as previously described. The samples were analyzed using nCounter PanCancer pathway panel (Nanostring technology #XT-CSO-PATH1-12, Seattle, WA, USA) according to the manufacturer's protocol. Quality control and normalization were performed using the R (version 3.6.1) package *NanoStringNorm* (version 1.2.1). First, a background correction robust to false positives was applied (*mean.2sd* method) and to minimize the impact of outlier values the geometric mean of housekeeping values was used to normalize the genes. Genes without detectable level across samples were removed. Subsequently, the differential analysis for gene expression was performed based on a t-test. P-values reported were the Benjamini-Hochberg false discovery rate (FDR) adjusted p-values. Genes with a log2 (fold change) < -0.58 or > 0.58 and a FDR < 0.05 were considered as differentially expressed.

Flow cytometry

Cell cycle analysis was performed in HCT116 cells treated with 100 μM GC7, 200 μM DFMO or 10 μM Cisplatin for 24h, as previously described[5]. DNA content was measured using C6, BD Accuri Flow Cytometer (BD Biosciences, San Jose, CA, USA) and analyzed using BD Accuri C6 software.

Annexin V-propidium iodide double staining was performed in HCT116 cells treated with 100 μM GC7, 200 μM DFMO, 10 μM Cisplatin for 48h and using the Annexin V Apoptosis Detection Kit FITC (#88-8005 e-Bioscience, Thermo Fischer Scientific), according to the manufacturer's instructions.

Immunohistochemistry

Immunohistochemistry was performed as described previously[6]. The following antibodies and conditions were used: anti Ki67 and anti Hypusine antibodies were diluted 1:100 in PBS solution and incubated overnight at four degrees. Signal was reveled using DAB substrate and DAB solution (Mouse to Mouse HRP Staining System-ScyTek laboratories, Logan, USA, HQ) according to the manufacturer's protocol. Sections were counterstained with hematoxylin.

Polyamines analysis

HCT116 cells were treated with 100 μM GC7 or vehicle for 72 hours. Polyamines content was determined by gas chromatography-mass spectrometry (GC-MS) as previously described [3]. Values were normalized to the protein concentration in the extract. The results are expressed as fold change, relative to untreated cells for each polyamine and represent the average ± SD of three independent experiments, each performed in triplicate.

MTT assay

MTT assay was performed as described previously[7]. HCT116 cells ($5x10^3$ cell/cm^2) were

plated in 96 multiwell plates. After 24 hours, cells were treated with 100 μM GC7, 200 μM DFMO or 10 μM Cisplatin for 48 hours. 0.5 mg/ml MTT solution (Sigma Aldrich) was added to the culture medium for 3h at 37°C. After the incubation, the medium was removed, 100 μl/well of DMSO were added and the absorbance was detected at 600 nm, using Glomax Explorer Multimode Microplate Reader (#GM3500 Promega).

SUPPLEMENTARY FIGURE LEGENDS

Supplementary Fig.1: Inhibition of polyamines/hypusine axis impairs CRC cell proliferation *in vitro*

A) HCT116 cells treated with 100 μM GC7 for 72 hours. Putrescine (PUT), Spermidine (SPD) and Spermine (SPM) levels were analyzed by GC-MS. Values were normalized by the protein concentration and expressed as fold change relative to vehicle control.
B) Hyp-EIF5A after 72h of GC7 treatments (decrescendo bars: 100 μM, 10 μM, 1 μM, 0.1 μM) from main Fig. 1a. Vinculin, loading control.
C) Growth curves of HT29 (*n*=3), HCT116, (*n*=3) SW480 (*n*=3), LoVo (*n*=3) cells, treated with the indicated concentrations of DFMO.
D) Hyp-EIF5A after 72h of DFMO treatments (decrescendo bars: 5 mM, 1 mM, 500 μM, 200 μM, 100 μM) as indicated. Vinculin, loading control.

For Statistical analysis: **p < 0.01, ***p < 0.001, ns not significant, by one-way ANOVA. Data represented the mean ± SD.

Supplementary Fig. 2: Heatmap of nanostring gene expression analysis from shDHPS vs SCR HCT116 cells

A) Heatmap showing the first 25 upregulated and first 25 downregulated genes. The row and column dendrograms represent the clustering using the hierarchical clustering method with the Pearson correlation as the similarity measure. Each row indicates z-score scaled expression levels.

Supplementary Fig. 3: DHPS ablation does not affect MYC mRNA turnover or its protein stability

A) Top, MYC RNA levels in HCT116 cells transduced for 72h with lentiviruses expressing shDHPS or non-specific shRNA (SCR); Data were normalized by L32. Bottom, Western blot showing corresponding Hyp-EIF5A and EIF5A. Vinculin, loading control (Bottom panel).
B) MYC, Hyp-EIF5A and EIF5A in HCT116 cells transduced for 72h with lentiviruses expressing shDHPS or non-specific shRNA (SCR), selected with puromycin for 72h and incubated with 100 μg/ml CHX for the indicated times. Vinculin, loading control. Densitometric analysis of MYC/Vinculin protein levels is shown at the bottom.

For Statistical analysis: ns not significant, by Student's *t* test. Data represented the mean ± SD

Supplementary Fig. 4: blockade of EIF5A-Hyp axis downregulates MYC

A) Hyp-EIF5A in HCT116 cells transduced for 72h with lentiviruses expressing shDHPS or non-specific shRNA (SCR) and selected with puromycin for 72h before transfection with WT and mutant MYC plasmids described in main Figure 4j. Vinculin, loading control
B) MYC, Hyp-EIF5A and EIF5A in HT29 treated with 100 μM GC7 for 72 hours. Vinculin, loading control.
C) Endogenous MYC isoforms (67 kDa and 64 kDa) in HCT116 cells transduced with lentiviruses expressing shEIF5A or non-specific shRNA (SCR) and selected with puromycin for 72h. Hyp-EIF5A, EIF5A and Vinculin (loading control) shown.
D) Endogenous MYC isoforms (67 kDa and 64 kDa) in HCT116 cells treated with 100 μM GC7 or CTRL vehicle for 24h. Hyp-EIF5A, EIF5A and Vinculin (loading control) shown.

REFERENCES

1. Canettieri G, Di Marcotullio L, Greco A, Coni S, Antonucci L, Infante P*, et al.* Histone deacetylase and Cullin3-REN(KCTD11) ubiquitin ligase interplay regulates Hedgehog signalling through Gli acetylation. *Nat Cell Biol*, **12**(2)**:** 132-142 (2010).

2. Dyer BW, Ferrer FA, Klinedinst DK, Rodriguez R. A noncommercial dual luciferase enzyme assay system for reporter gene analysis. *Analytical biochemistry*, **282**(1)**:** 158-161 (2000).

3. D'Amico D, Antonucci L, Di Magno L, Coni S, Sdruscia G, Macone A*, et al.* Non-canonical Hedgehog/AMPK-Mediated Control of Polyamine Metabolism Supports Neuronal and Medulloblastoma Cell Growth. *Dev Cell*, **35**(1)**:** 21-35 (2015).

4. Barczak W, Suchorska W, Rubis B, Kulcenty K. Universal real-time PCR-based assay for lentiviral titration. *Molecular biotechnology*, **57**(2)**:** 195-200 (2015).

5. Coni S, Mancuso AB, Di Magno L, Sdruscia G, Manni S, Serrao SM*, et al.* Selective targeting of HDAC1/2 elicits anticancer effects through Gli1 acetylation in preclinical models of SHH Medulloblastoma. *Scientific reports*, **7:** 44079 (2017).

6. Di Magno L, Manni S, Di Pastena F, Coni S, Macone A, Cairoli S*, et al.* Phenformin Inhibits Hedgehog-Dependent Tumor Growth through a Complex I-Independent Redox/Corepressor Module. *Cell reports*, **30**(6)**:** 1735-1752 e1737 (2020).

7. Antonucci L, Di Magno L, D'Amico D, Manni S, Serrao SM, Di Pastena F*, et al.* Mitogen-activated kinase kinase kinase 1 inhibits hedgehog signaling and medulloblastoma growth through GLI1 phosphorylation. *Int J Oncol*, **54**(2)**:** 505-514 (2019).

Supplementary Fig. 1

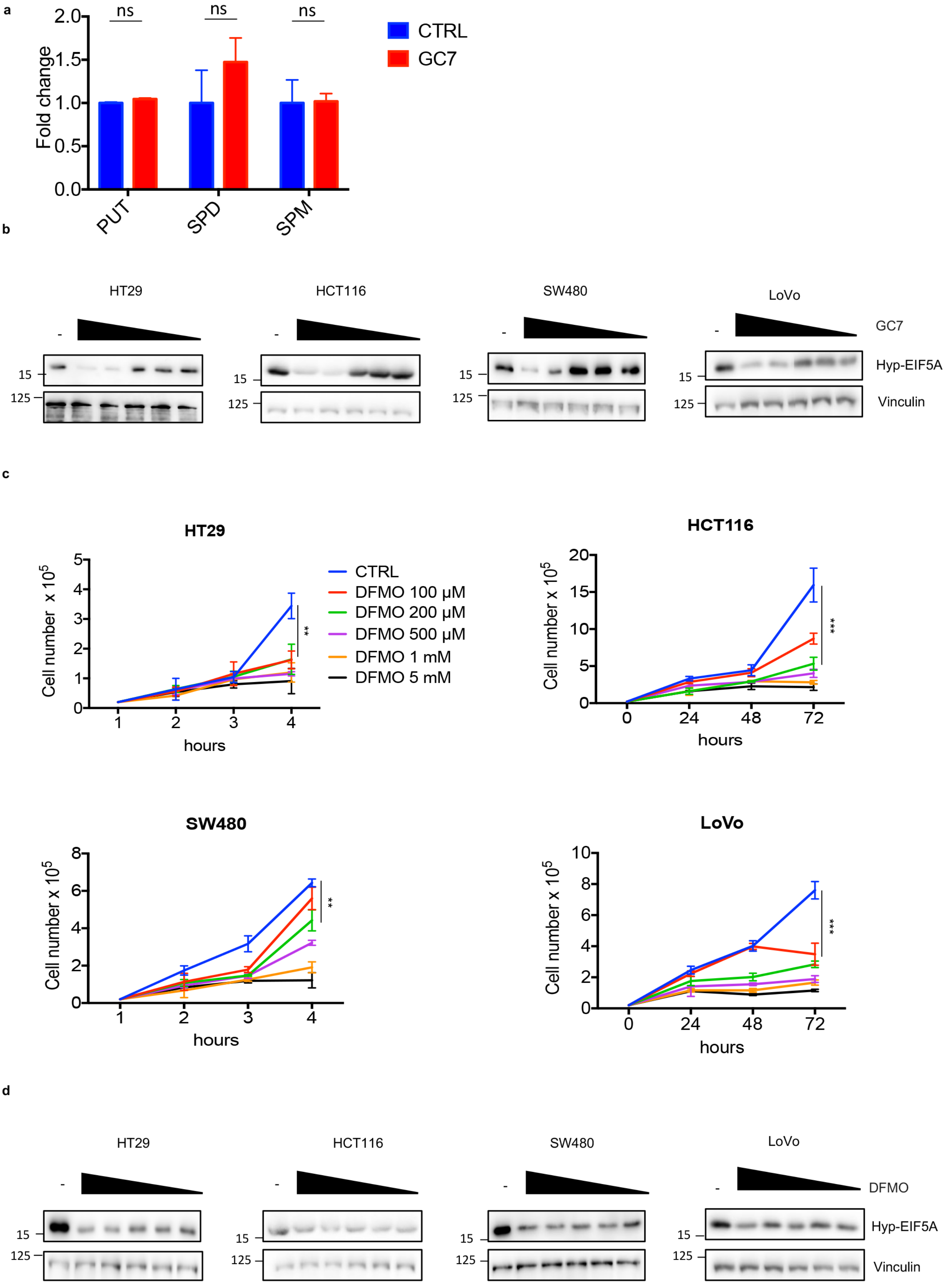

Supplementary Fig. 2

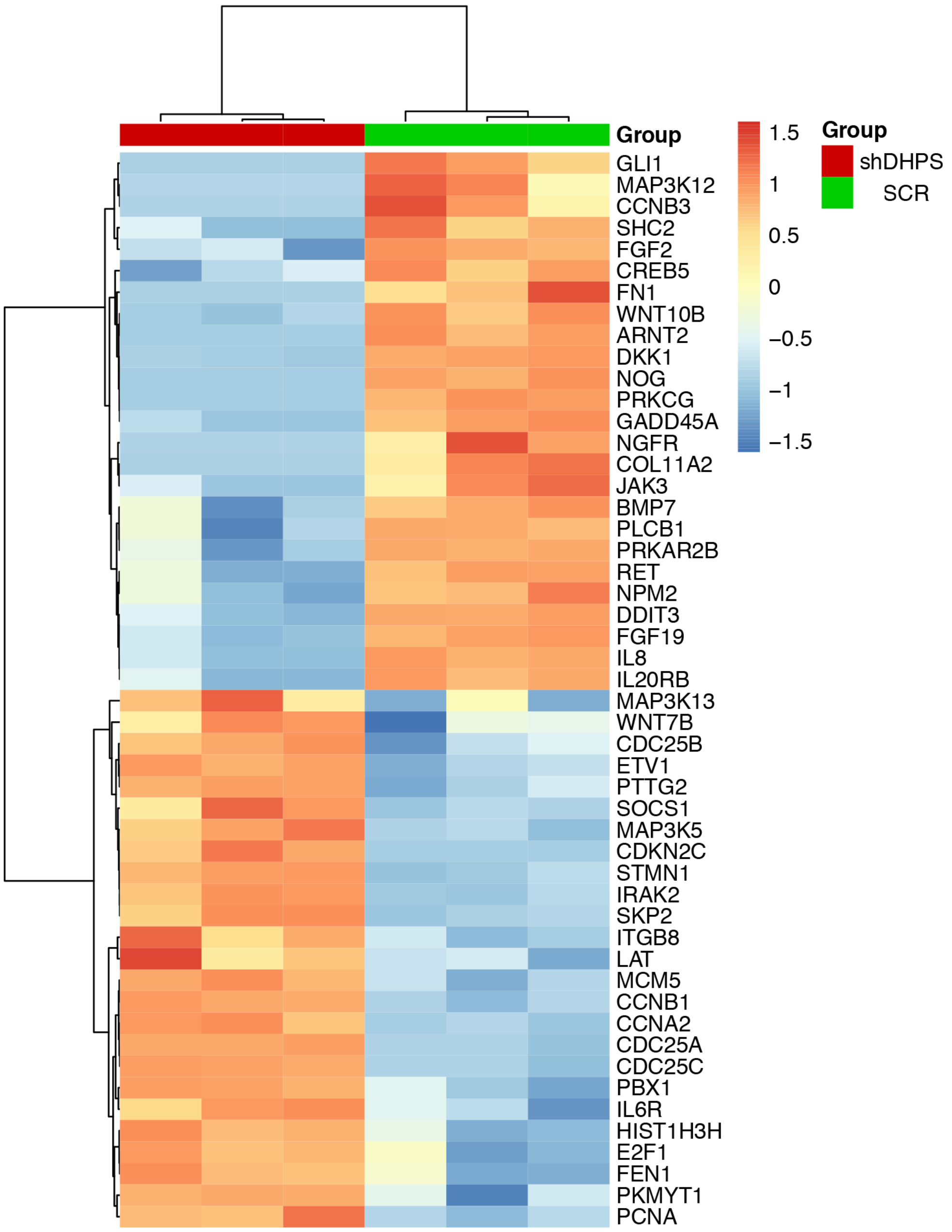

Supplementary Fig. 3

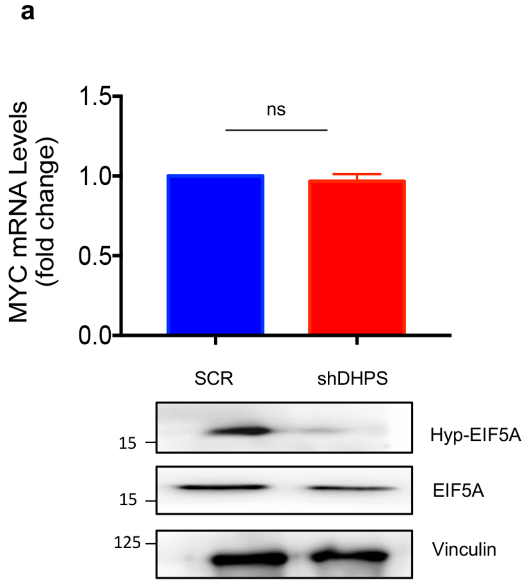

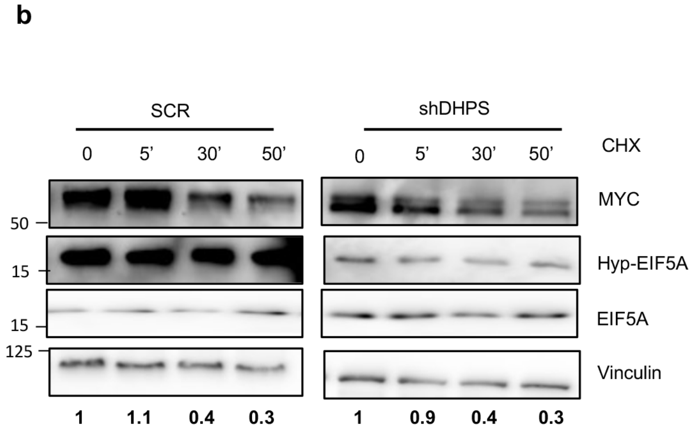

Supplementary Fig. 4

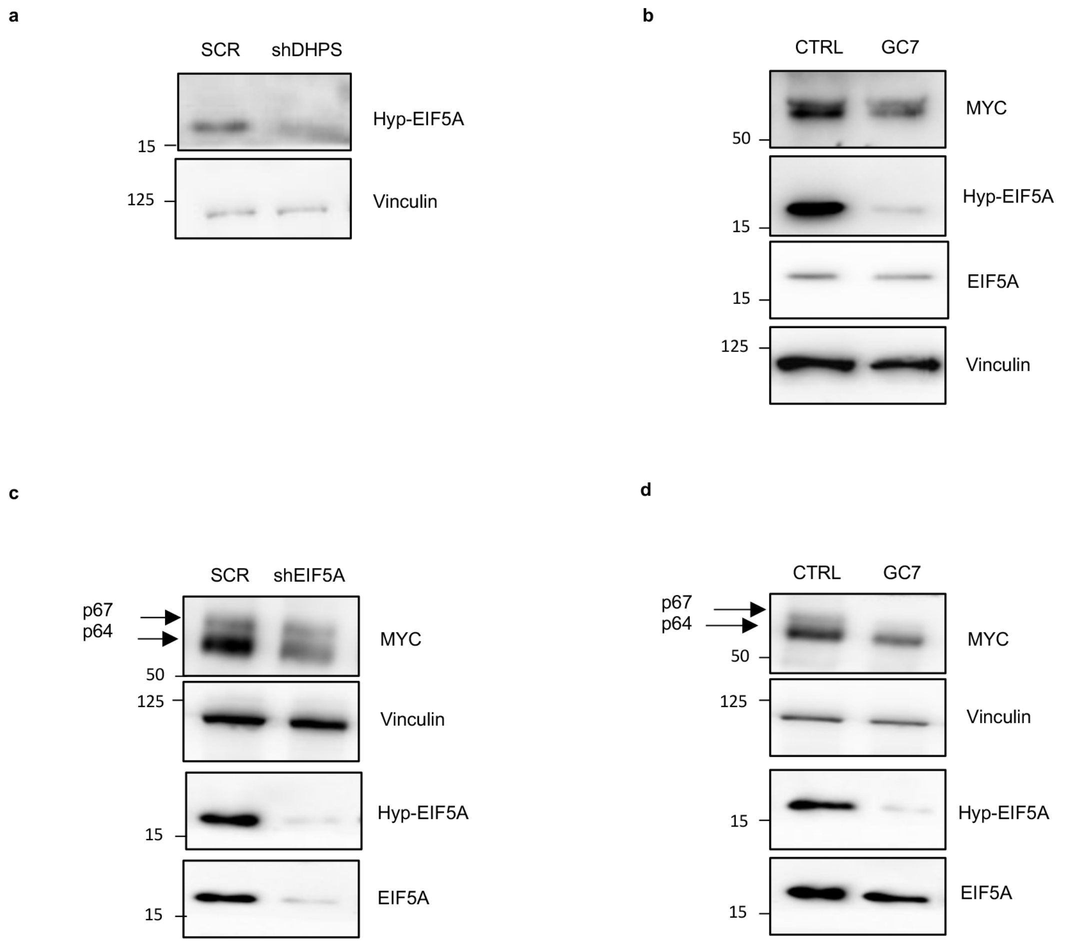

Supplement: Supplementary file 1 — Supplemental material [file 41419_2020_3174_MOESM1_ESM.pdf]
